# Associations between postpartum pain, mood, and maternal–infant attachment and parenting outcomes

**DOI:** 10.1038/s41598-022-21793-1

**Published:** 2022-10-24

**Authors:** Mutasim Makeen, Lia M. Farrell, Kelsea R. LaSorda, Yangyang Deng, Valeria Altamirano, Olivia Jarvis, Tanya Kenkre, Grace Lim

**Affiliations:** 1 Department of Anesthesiology and Perioperative Medicine, University of Pittsburgh, Pittsburgh, PA USA; 2 Department of Obstetrics and Gynecology, UPMC Magee-Womens Hospital, University of Pittsburgh School of Medicine, 300 Halket Street #3510, Pittsburgh, PA 15215 USA

**Keywords:** Psychology, Medical research

## Abstract

Pain and depression are interrelated, and worse postpartum pain has been associated with postpartum depression. It remains unclear whether improved pain and mood after delivery can also improve maternal parenting. Few studies have examined relationships between postpartum pain and negative mood (anxiety or depression) or their effects on parent–infant relationship outcomes. The purpose of this study was to explore the relationships between postpartum pain, mood, parent–infant attachment, parenting self-efficacy, and infant development. This was a prospective longitudinal observational pilot study of nulliparous women enrolled at the third trimester and presenting for labor and delivery at term gestation. Baseline third trimester assessments included validated inventories of pain (the brief pain inventory, BPI), depression (the Edinburgh postnatal depression screen, EPDS), anxiety (the state trait anxiety inventory, STAI), multidimensional scale of perceived social support (perceived social support scale, MSPSS) and perceived stress scale (PSS). Demographic and labor characteristics were recorded. At 6 weeks and 3 months postpartum, self-reported assessments included EPDS, STAI, BPI, maternal parent infant attachment scale (MPAS), and perceived maternal parenting self-efficacy (PMP-SE). Child development outcomes were assessed at 6 weeks and 3 months using the Ages and Stages Questionnaire (ASQ). Univariable linear regression assessed the relationships between pain and parenting outcomes (MPAS and PMP-SE), including potential interactions between pain and mood for parenting outcomes. Generalized linear modeling was used to explore the relationships between postpartum pain, parenting outcomes, and child development outcomes. Of 187 subjects, 87 had complete data on parent–infant attachment and parenting self-efficacy data at 3 months. Lower "pain right now" scores (BPI) on postpartum day 1 was associated with higher maternal–infant attachment (MPAS) at 6 weeks postpartum (Estimate − 1.8, 95% CI − 3.4 to − 0.2, *P* < 0.03) but not at 3 months (Estimate 0.23 95% CI − 1.1 to 1.6, *P* = 0.7). Higher depression (EPDS) scores at 6 weeks were also associated with lower MPAS scores at 6 weeks (Estimate − 1.24, 95% CI − 2.07 to − 0.40, *P* = 0.004). However, there was no evidence that the relationship between pain and MPAS varied by depression score at 6 weeks (*P* = 0.42). Pain scores at baseline, six weeks, or three months did not correlate with parenting outcomes (MPAS, PMP-SE) at six weeks or three months. Results of the generalized linear modeling revealed relationships between pain, age, anxiety (STAI), and depression (EPDS) predictors, and the outcomes of parenting (MPAS, PMP-SE) and gross motor and personal–social (ASQ) aspects of infant development. There is a pattern of association between worse postpartum pain, anxiety, and depression with worse parenting outcomes. Depression and pain may also affect infant development, but future work is required to replicate and characterize these potential relationships.

## Introduction

Pain and depression are bi-directionally related, and worse postpartum pain has been linked to postpartum depression (PPD)^1, 2^. PPD is a significant public health problem, associated with complications ranging from to social isolation and economic ramifications from reducing work or leaving the workforce, to maternal suicide in its most severe cases^3^. PPD and postpartum anxiety are associated with parenting behavioral deficits including increased aggression, lack of communication, and poor responsiveness, all of which harm the parent–caregiver role^4, 5^. PPD also negatively impacts breastfeeding duration, parent and infant sleeping patterns, and introduction of solid foods, which can affect infant neurocognitive development and growth^6–8^. Offspring of mothers with PPD are at risk for impaired attachment, impaired cognitive development, conduct disorders, and depression in adolescence^9^.

The significant burden on patients and communities makes modifiable risk factors for PPD an important area of research. Prior studies have suggested that among other factors like pre-existing anxiety and poor social support, risk for PPD may be influenced by pain. Specifically, PPD risk may be increased by individual experience and perception of severe acute pain, rather than the degree of tissue damage^10–12^. Our research has also suggested a relationship between postpartum pain and psychosocial variables (e.g., perceived social support, perceived stress)^13^. However, to our knowledge, the influence of postpartum pain on not only depression, but also parenting and infant behavioral outcomes, have not been rigorously investigated.

The primary goal of this study was to assess the relationships between postpartum pain, maternal mood, and parenting self-efficacy and maternal–infant attachment. Secondarily, we aimed to explore the relationships between postpartum pain, maternal psychosocial variables, and child development.

## Methods

The University of Pittsburgh Institutional Review Board approved the study (PRO15030338) and written informed consent was obtained from all participants. All methods were conducted in accordance with relevant guidelines and regulations including CONSORT 2010 guidelines. Data supporting the findings of this study are available from the corresponding author, but restrictions apply to the availability of these data which were used under license for the current study so are not publicly available. The datasets used and analyzed during the current study available from the corresponding author on reasonable request.

### Patients

Pregnant women receiving perinatal care at the University of Pittsburgh Medical Center Magee-Women’s Hospital, or The Midwife Center for Birth and Women’s Health (Pittsburgh, PA, USA) were screened and approached if eligible at their third trimester prenatal clinic visit. After providing written informed consent, women planning on spontaneous labor or scheduled induction of labor at term gestation were enrolled. Inclusion criteria were presentation to our labor and delivery unit at ≥ 38 weeks gestation and complete baseline inventories. Exclusion criteria were age < 18, non-English speaking as surveys were validated in the English language, chronic pain history, taking medication for opioid use disorder, severe obstetric disease (i.e., pre-eclampsia or eclampsia requiring magnesium therapy, necessity for immediate cesarean delivery without labor), class 3 obesity with body mass index ≥ 40 kg/m^2^, fetal anomalies or growth restriction, contraindications to neuraxial anesthesia, inability to follow the study protocol for 3 months, admission for labor and delivery not at term gestation, and plans for newborn adoption.

### Demographic and obstetric data and patient-reported instruments

Self-reported demographic characteristics were completed, and obstetric and labor characteristics were collected from the medical record. Variables included race, ethnicity, mode of delivery, epidural analgesia utilization in labor. We also recorded history of abuse (i.e., partner, sexual, domestic, and childhood abuse), as well as histories of substance abuse, anxiety, depression, and other mental illness. Participants electronically completed prenatal assessments of depression (Edinburgh Postnatal Depression Scale, EPDS), anxiety (State-Trait Anxiety Inventory, STAI)^2, 14^, resiliency (Ego-Resiliency Scale, ER-89)^15^, and pain catastrophizing (Pain Catastrophizing Scale, PCS)^16^, multidimensional scale of perceived social support (MSPSS), and a pain inventory (Brief Pain Inventory—Long Form, BPI-L)^17^. These variables were selected based on validated associations with pain, depression, or both^17, 18^. Pain (BPI Short Form, BPI-S), and perceived stress scale (PSS) were recorded on the first or second postpartum day. At six weeks postpartum, participants completed the EPDS, BPI-S, the maternal parent attachment scale (MPAS), the parenting self-efficacy (PMP-SE), and the Ages and Stages Questionnaire (ASQ) for two months (ASQ-2 months). At three months postpartum, participants were administered the EPDS, the BPI-S, the ASQ-4 months. The ASQ-2 months and -4 months were used at 6 weeks and 3 months respectively. The four timepoints considered were baseline and postpartum day 1, 6 weeks postpartum, and 3 months postpartum.

EPDS is a self-completed, 10-item scale developed specifically for women in the perinatal period. It was shown to identify patients at risk for perinatal depression^1, 19^. It is essential to note that EPDS not intended to substitute for a mental health professional’s diagnosis of depression; it has estimated 80% sensitivity for the diagnosis^1, 19^. Nevertheless, because it is not as specific as other instruments, the EPDS might identify some women who are not depressed. Therefore, the scale should be used to screen patients appropriate for specialist referral^1, 20^. The State-Trait Anxiety Inventory (STAI) is a psychological inventory consisting of 40 self-report items on a 4-point Likert scale. The STAI measures two types of anxiety—state anxiety and trait anxiety. Higher scores are positively correlated with higher levels of anxiety. The Brief Pain Inventory—Short Form (BPI-SF) is a 9-item self-administered questionnaire used to evaluate the severity of a patient's pain and the impact of this pain on the patient's daily functioning. The Perceived Stress Scale (PSS) is a stress assessment instrument consisting of 10 items with stress-related questions. The Pain Catastrophizing Scale (PCS) assesses the extent of catastrophic thinking. It is a 13-item scale, with a total range of 0 to 52. Higher scores are associated with higher amounts of pain catastrophizing. The Maternal Postnatal Attachment Scale (MPAS) is a 19-item self-report questionnaire that is used to assess mother-to-infant attachment. Maternal Parenting Self-Efficacy (PMP S-E) has 20 items rated with a four-point scale ranging from 1 (strongly disagree) to 4 (strongly agree). Higher scores indicate higher levels of perceived self-efficacy. The Ages and Stages Questionnaires (ASQ) are composed of 5 components each has 6 questions completed by parents, evaluating infant and child development.

### Statistical analysis

The primary predictor of interest was “pain right now” assessed by the BPI on a 0–10 numeric rating scale where 0 is “no pain” and 10 is “pain as bad as you can imagine” (corresponding questions #15 on the BPI-L at baseline and #6 on the BPI-S at postpartum days 1, 6 weeks, and 3 months). The primary outcomes were MPAS and PMP-SE at 6 weeks and 3 months. Univariate regression explored the associations between the outcomes of MPAS and PMPSE at 6 weeks and 3 months, and the predictor variables for pain, mood (anxiety, STAI; depression, EPDS), epidural analgesia use, catastrophizing (PCS), social support (MSPSS), perceived stress (PSS), and resiliency. Interaction terms were assessed for any pain and mood predictor variables that met the significance threshold of P < 0.10 in univariate analyses.

#### Generalized linear model analysis

General linear modeling was used to explore the relationship between the primary predictor of “pain right now” at baseline (measured by the BPI) and other baseline covariates (age, perceived stress by MSPSS, anxiety by STAI, depression by EPDS), for the outcomes of parenting (maternal–infant attachment scale by MPAS and parenting self-efficacy by PMPSE) and child development (ages and stages questionnaire 3rd edition, ASQ-3) at 6 weeks and 3 months postpartum.

#### Sample size calculation

As a pilot study, we targeted 10% of the sample required for a full study that would evaluate primary relationships between postpartum pain and depression. The presumed baseline incidence of postpartum depression in American women was 10–20%. For a full study, based on previous work that compared non-epidural pain relief during delivery and postpartum depression, a sample size of 400 in each group (excessive pain versus limited pain) would have 80% power to detect a 10% difference in postpartum depression between groups with a significance level (alpha) of 0.05 (two-tailed). Accounting for an estimated dropout rate of 15%, we expected expect to enroll a total of 430 subjects in each group. The risk for postpartum depression was defined by a positive screen (score ≥ 10) on the EPDS tool. Therefore, we targeted a sample size of 86 patients, as would yield 10% of the sample size required for a full study.

Statistical analyses were performed using StataSE (version 17.1, College Station, TX), Excel (version 16.35, Microsoft), and SAS, version 9.4 statistical software (SAS Institute Inc., Cary, NC). Descriptive statistics were presented as means (standard deviations), medians (with 25th and 75th percentiles) for continuous data and as absolute frequencies and percentages for categorical data.

## Results

**Figure 1:**
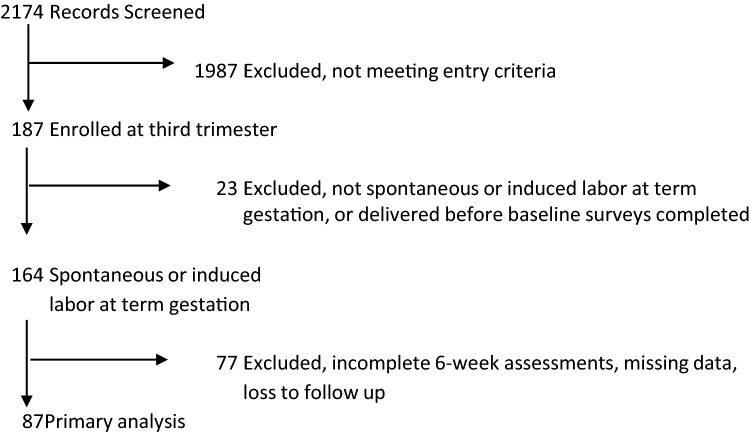
Study flow chart.

There were 187 enrolled participants, of whom 87 had complete data for analysis (Fig. 1). The characteristics of the study population are in Table 1. Univariable analysis compared parenting outcomes (i.e., MPAS and PMP-SE) by pain and mood variables at baseline, 6-week, and 3-month time points (Table 2). The univariable regression results for estimating the effect of postpartum pain score on 6-week and 3-month pain severity showed significant correlations between pain variables and MPAS and PMP-SE outcomes at 6 weeks and 3 months. Lower "pain right now" scores on postpartum day 1 were associated with higher maternal–infant attachment at 6 weeks (Estimate − 1.8, 95% CI − 3.4 to − 0.2, P < 0.03) but not at 3 months (Estimate 0.23 95% CI − 1.1 to 1.6, *P* = 0.7). Other pain assessments were not significantly associated with maternal–infant attachment or parenting self-efficacy outcomes at 6 weeks or 3 months. Baseline depression, resiliency, and perceived stress scores on day 1 were associated with maternal–infant attachment at 3 months. Pain catastrophizing and resiliency at baseline were associated with parenting self-efficacy at 3 months (Table 2).

**Table 1:** Baseline study population characteristics and scored results of the instruments.

	Result (N = 187)
Age—years	30 [4.8]
Epidural labor analgesia utilization rate—yes/no	116/187 (62.0%)
**Race**	
White	142/187 (75.9%)
Black/African American	41/187 (21.9%)
Asian	14/187 (7.4%)
Hawaiian	1/187 (0.5%)
American Indian/Alaskan	4/187 (2.13%)
Other	2/187 (1%)
**Ethnicity**	
Hispanic	8/187 (4.3%)
Non-Hispanic	179/187 (95.7%)
**Mode of delivery (N = 147)**	
Normal spontaneous vaginal delivery	103/144 (71.5%)
Cesarean delivery—arrest of dilation or descent	15/144 (10.4%)
Cesarean delivery—other reason	9/144 (6.25%)
Cesarean delivery—non-reassuring fetal status	7/144 (4.8%)
Assisted vaginal delivery	5/144 (3.74%)
**History of abuse—yes/no**	10/187 (5.2%)
Partner	2/187 (1.3%)
Sexual	5/187 (2.6%)
Domestic	2/187 (1.3%)
Childhood	10/187 (5.1)
Other	0/187 (0%)
History of substance abuse—yes/no	0/187 (0%)
History of anxiety or depression—yes/no	59/187 (31.6%)
**History of other mental illness**	15/187 (7.8%)
Bipolar disorder	7/187 (3.9%)
Schizophrenia	0/187 (0%)
Post-traumatic stress disorder	2/187 (1.3%)
Attention deficit hyperactivity disorder	2/187 (1.3%)
Other	7/187 (3.9%)
**Inventory results by time**	
Baseline	
STAI	79.1 [28.1]
PCS	10.0 [8.5]
ER89	36.9 [16.4]
BPI Long	2.2 [2.2]
EPDS	4.4 [3.7]
Day 1	
PSS	13.2 [5.5]
BPI Short #6	3.1 [2.2]
Postpartum 6 weeks	
BPI Short #6	0.6 [1.6]
EPDS	4.4 [4.1]
PMP-SE	53.3 [26.6]
MPAS	46.8 [15.6]
ASQ Gross Motor	7.6 [2.0]
ASQ Personal Social	8.9 [2.2]
Postpartum 3 months	
BPI Short #6	0.6 [1.5]
EPDS	4.1 [3.8]
PMP-SE	48.7 [30.4]
MPAS	49.5 [12.3]
ASQ gross motor	7.3 [1.5]
ASQ personal social	8.5 [2.4]

The population consisted of healthy, nulliparous pregnant women (aged ≥ 18 years) at term gestation.

presenting for either spontaneous or induced labor and delivery at ≥ 38 weeks estimated gestational age.

*BPI* brief pain inventory, *STAI* state trait anxiety inventory, *EPDS* Edinburgh postnatal depression scale, *PMP-SE* perceived maternal parenting self-efficacy, *MPAS* maternal postnatal attachment scale, *PSS* perceived social support, *ASQ* ages and stages questionnaire, *PCS* pain catastrophizing scale, *ER89* ego resiliency-89 scale.

Data are presented as frequency (percentage) or mean [standard deviation].

**Table 2:** Univariate analyses identifying predictors of maternal–infant attachment (MPAS) and perceived maternal parenting self-efficacy (PMPSE) at 6 weeks and 3 months postpartum.

**MPAS 6 weeks**				
Variable (units)	R^2^	Estimate	Confidence Interval	P-value
Pain right now baseline	0.13	− 2.22	− 4.69 to 0.25	0.08
Pain right now postpartum day 1	0.061	− 1.8	− 3.44 to − 0.16	0.032*
Pain right now 6 weeks	0.009	− 0.92	− 3.19 to 1.34	0.42
STAI baseline	0.02	0.07	− 0.05 to 0.20	0.25
EPDS baseline	0.02	− 0.53	− 1.51 to 0.45	0.28
EPDS 6 weeks	0.10	− 1.24	− 2.07 to − 0.40	0.004*
Epidural use	0.004	1.92	− 5.42 to 9.27	0.64
History of anxiety or depression	0.001	− 2.52	− 10.27 to 5.24	0.52
History of mental illness	0.006	− 2.52	− 10.27 to 5.24	0.52
ER89 baseline	0.001	0.03	− 0.19 to 0.25	0.78
PCS baseline	0.0004	− 0.038	− 0.46 to 0.38	0.86
MSPSS baseline	0.001	− 0.02	− 0.16 to 0.12	0.78
PSS postpartum day 1	0.017	− 0.37	− 1.02 to 0.28	0.26

**MPAS 3 months**				
Variable (units)	R^2^	Estimate	Confidence Interval	P-value
Pain right now baseline	0.001	− 0.31	− 3.85 to 3.24	0.86
Pain right now postpartum day 1	0.002	0.24	− 1.09 to 1.56	0.72
Pain right now 6 weeks	0.007	0.62	− 1.16 to 2.41	0.49
Pain right now 3 months	0.0004	0.14	− 1.61 to 1.90	0.87
STAI baseline	0.003	0.026	− 0.08 to 0.13	0.62
EPDS baseline	0.07	− 0.88	− 1.62 to − 0.13	0.022*
EPDS 6 weeks	0.007	− 0.25	− 0.94 to 0.44	0.48
EPDS 3 months	0.003	− 0.18	− 0.92 to 0.57	0.64
Epidural use	0.005	− 1.82	− 7.61 to 3.97	0.53
History of anxiety or depression	0.012	− 2.73	− 8.82 to 3.35	0.37
History of mental illness	0.024	− 6.96	− 17.35 to 3.44	0.19
ER89 baseline	0.103	0.239	0.08 to 0.40	0.005*
PCS baseline	0.013	− 0.16	− 0.49 to 0.17	0.33
MSPSS baseline	0.006	0.04	− 0.08 to 0.16	0.5
PSS postpartum day 1	0.083	− 0.064	− 1.12 to − 0.14	0.012*

**PMP-SE 6 weeks**				
Variable (units)	R^2^	Estimate	Confidence Interval	P-value
Pain right now baseline	0.00	− 0.03	− 5.38 to 5.33	0.99
Pain right now postpartum day 1	0.0002	− 0.18	− 3.05 to 2.69	0.90
Pain right now 6 weeks	0.014	− 1.99	− 5.83 to 1.86	0.31
STAI baseline	0.004	0.06	− 0.16 to 0.27	0.61
EPDS baseline	0.0004	− 0.15	− 1.81 to 1.50	0.86
EPDS 6 weeks	0.023	0.99	− 0.51 to 2.49	0.19
Epidural use	0.002	2.13	− 10.54 to 14.81	0.74
History of anxiety or depression	0.007	4.68	− 8.55 to 17.92	0.48
History of mental illness	0.19	− 16.4	− 40.93 to 8.13	0.19
ER89 baseline	0.012	0.18	− 0.19 to 0.56	0.33
PCS baseline	0.011	0.32	− 0.40 to 1.03	0.38
MSPSS baseline	0.03	0.17	− 0.07 to 0.41	0.16
PSS postpartum day 1	0.0003	0.08	− 1.03 to 1.20	0.89

**PMP-SE 3 months**				
Variable (units)	R^2^	Estimate	Confidence Interval	P-value
Pain right now baseline	0.001	0.41	− 5.47 to 6.29	0.89
Pain right now postpartum day 1	0.0002	− 0.18	− 3.46 to 3.10	0.91
Pain right now 6 weeks	0.003	1.06	− 3.35 to 5.48	0.63
Pain right now 3 months	0.0001	− 0.20	− 4.96 to 4.57	0.93
STAI baseline	0.018	0.15	− 0.11 to 0.41	0.25
EPDS baseline	0.015	1.00	− 0.91 to 2.90	0.30
EPDS 6 weeks	0.003	0.37	− 1.34 to 2.09	0.67
EPDS 3 months	0.004	− 0.56	− 2.40 to 1.28	0.55
Epidural use	0.005	4.57	− 9.81 to 18.94	0.53
History of anxiety or depression	0.0003	− 1.02	− 16.05 to 14.00	0.89
History of any mental illness	0.0002	− 1.39	− 27.22 to 24.44	0.92
ER89 baseline	0.06	− 0.44	− 0.85 to − 0.02	0.04*
PCS baseline	0.061	0.88	0.08 to 1.68	0.03*
MSPSS baseline	0.003	− 0.07	− 0.35 to 0.22	0.65
PSS postpartum day 1	0.015	0.66	− 0.60 to 1.93	0.30

*P < 0.05.

*MPAS* maternal parent infant attachment scale, *PMP-SE* perceived maternal parenting self-efficacy scale, *STAI* state trait anxiety inventory, *EPDS* Edinburgh postnatal depression scale, *ER89* ego resiliency scale, *PCS* pain catastrophizing scale, *MSPSS* multidimensional scale of perceived social support scale, *PSS* perceived stress scale.

### Interactions between pain and mood for parenting outcomes

The only association between pain and parenting outcome was noted between “pain right now” on postpartum day 1 and MPAS at 6 weeks. Univariable regression also revealed an association between baseline depression (EPDS) and MPAS at 6 weeks (Table 2). To assess if the association between “pain right now” on postpartum day 1 and MPAS at 6 weeks was different according to depression score at 6 weeks, we assessed the interaction term between depression and pain. There was no evidence that the relationship between pain and MPAS varied by depression score at 6 weeks (Estimate 0.14, 95% CI − 0.20 to 0.47, *P* = 0.422).

### Generalized linear model results

Child Development Outcomes (ASQ-3): Baseline “pain right now” scores were associated with ASQ gross motor scores at 3 months (Estimate 1.25, R^2^ = 0.929 and *P* < 0.001). EPDS at baseline was a significant predictor for ASQ personal social scores at 3 months (Estimate 1.875, R^2^ = 0.804 and *P* < 0.05) (Table 3).

**Table 3:** Exploratory analysis between pain and outcomes of interest using generalized linear modeling. The primary predictor of interest was from the Brief Pain Inventory (“pain right now” on a 0–10 numeric rating scale) at baseline. The results suggest that pain is not associated with parenting attachment, parenting self-efficacy. The results suggest that there may be an association between baseline pain and infant gross motor development scores, although the accuracy of the model fit was not significant. *BPI* Brief Pain Inventory, Short Form, *MPAS* Maternal Parent Attachment Scale, *ASQ-3* Ages and Stages Questionnaire, 3rd Edition, *PMP-SE* parenting self-efficacy scale, *MSPSS* Multidimensional Scale of Perceived Social Support, *STAI* state-trait anxiety inventory, *EPDS* Edinburgh postnatal depression scale, *EST* estimate, *STD* standard deviation.

Outcome	MPAS				ASQ-3								PMP-SE			
					Gross motor				Personal social							
Period	6 Weeks		3 Months		6 Weeks		3 Months		6 Weeks		3 Months		6 Weeks		3 Months	
Predictor	EST (STD)	R^2^	EST (STD)	R^2^	EST (STD)	R^2^	EST (STD)	R^2^	EST (STD)	R^2^	EST (STD)	R^2^	EST (STD)	R^2^	EST (STD)	R^2^
**Primary predictor**																
BPI (Short #6)	− 0.22 (0.142)	0.029	− 0.118 (0.133)	0.010	− 0.154 (0.294)	0.005	1.25 (0.314)***	0.929	− 0.083 (0.204)	0.002	0.139 (0.897)	0.008	− 0.375 (0.366)	0.013	− 0.133 (0.342)	0.002
**Other baseline covariates**																
Age	− 0.177 (0.075)*	0.067	− 0.173 (0.073)*	0.072	− 0.096 (0.167)	0.007	− 0.561 (0.442)	0.617	0.13 (0.104)	0.022	− 1.06 (0.51)	0.590	− 0.326 (0.195)	0.035	− 0.188 (0.191)	0.013
MSPSS	0.032 (0.025)	0.019	0.025 (0.023)	0.016	− 0.074 (0.066)	0.024	0.24 (0.534)	0.168	− 0.019 (0.035)	0.004	− 0.36 (0.526)	0.190	0.042 (0.067)	0.005	0.054 (0.059)	0.011
STAI	0.053 (0.038)	0.022	0.054 (0.035)	0.029	0.02 (0.074)	0.001	0.357 (1.856)	0.036	− 0.002 (0.056)	0.000	− 0.14 (0.431)	0.034	0.254 (0.1)*	0.073	0.199 (0.09)*	0.059
EPDS	− 0.192 (0.089)*	0.052	− 0.201 (0.089)*	0.062	0.416 (0.209)	0.071	0.921 (0.684)	0.645	− 0.017 (0.142)	0.000	1.875 (0.535)*	0.804	− 0.658 (0.255)*	0.075	− 0.524 (0.229)*	0.063

**Note that bolded P value indicated statistical significance at 95% confidence interval, i.e., *0.01 < = P < 0.05, **0.001 < = P < 0.01, ***P < 0.001.

***P-value indicates statistical significance at 95% Confidence Interval regarding each covariate to the outcome; R^2^ reflects the accuracy of the model fit.

Parenting Outcomes (MPAS and PMP-SE): Age was a significant predictor of MPAS at 6 weeks (Estimate − 0.177, R^2^ = 0.067 and *P* < 0.05) and at 3 months (Estimate − 0.173, R^2^ = 0.072 and *P* < 0.05) and EPDS at baseline is also a significant predictor of MPAS at 6 weeks (Estimate − 0.192, R^2^ = 0.052 and *P* < 0.05) and at 3 months (Estimate − 0.201, R^2^ = 0.062 and *P* < 0.05). STAI at baseline is a predictor of PMP-SE at 6 weeks (Estimate 0.254, R^2^ = 0.073 and *P* < 0.05) and at 3 months (Estimate 0.199, R^2^ = 0.059 and *P* < 0.05). Finally, EPDS at baseline is a significant predictor of PMP-SE at 6 weeks (Estimate − 0.658, R^2^ = 0.075 and *P* < 0.05) and at 3 months (Estimate − 0.524, R^2^ = 0.063 and *P* < 0.05) (Table 3).

## Discussion

The main findings of this prospective observational study are that there are patterns of association between postpartum pain, mood, psychosocial variables, and parenting outcomes. Our results are hypothesis generating and suggest that acute pain, depression, resiliency, perceived stress, and pain catastrophizing might play a role in maternal–infant attachment, parenting self-efficacy, and infant development. These findings urge replication in a larger sample size and help contribute to filling a knowledge gap regarding the effects of pain and mood on maternal–infant attachment as well as outcomes related to the parent–infant dyad.

Previous studies have investigated the relationships between pain symptoms and self-efficacy on mood and function. Drenkard et al. study found links between pain symptom self-efficacy, pain interference, and depression in patients with systemic lupus^21^. Similar to our findings, age was associated with these relationships, modifying the effect of self-efficacy on pain interference. Women ≥ 55 years old who had low symptom self-efficacy reported disproportionately higher pain interference with daily activities compared with women < 35 years old. Similarly, Cheng et al. reported relationships between pain self-efficacy, pain catastrophizing, pain intensity, and depression^22^. Our study adds to the existing knowledge on how worse postpartum pain, alongside other important mood, and personality factors, can contribute to poor parenting and infant outcomes. Potential reasons why pain may affect parenting outcomes and infant development, may be due to the effects of pain interference and impaired mental and physical functioning on successful emotional attachments and fulfilling the parenting role. These impairments may have subsequent deleterious effects on infant neurocognitive development. The correlation with these types of relationships and parenting-infant outcomes are worth investigating in the future.

We found a relationship between baseline anxiety and parenting self-efficacy scores at 6 weeks postpartum, and between baseline “pain right now” and infant gross motor development at 3 months. Perinatal anxiety is a maladaptive behavioral condition, and its long-term effects on maternal functioning and infant development represent serious psychological and physical health consequences^23^. Further, anxiety and depression symptoms often co-occur, and the presence of the co-morbidity is a marker of severity^24, 25^. Our study findings point to potential relationships between maternal pain, depression and anxiety, and the gross motor component childhood development. Future work with a larger sample would clarify these potential relationships.

This study has limitations. The scales that were used in this pilot study were intended as screening instruments and do not necessarily indicate a clinical diagnosis of any mood disorder or provide objective measures of child development and attachment. As an observational study, one cannot draw cause and effect conclusions regarding the associations detected in this analysis. Because we did not assess the impact of pain on physical or mental function, for example, it is not possible to provide data supporting a potential mechanism for why pain may have had measurable effects on MPAS. Mode of delivery is known to influence postpartum pain with cesarean delivery inducing greater risk for severe postpartum pain. However, due to sample size and the primary study question focused on pain itself, we did not account for mode of delivery as a co-variate in our analyses; however, we believe the current findings are compelling to support and justify future research that accounts for the anticipated major differences in postpartum pain among people experiencing cesarean delivery vs. vaginal delivery. The exclusion of Class 3 obesity limits any generalizability to this population. The use of both state and trait anxiety subscales in this analysis was due to a desire to avoid model collinearity, but that approach may have limited the ability to detect relationships between the subscales on the variables of interest. A future study with a larger sample size should specifically investigate pain interference, pain self-efficacy, and depression, because pain interference has been linked to poor psychosocial outcomes in patients with chronic pain.

## Conclusion

We observe a pattern of association between worse postpartum acute pain and anxiety/depression after birth, with worse parenting outcomes including maternal infant attachment and parenting self-efficacy, and with infant development at 6 weeks and 3 months. The relationships between pain and maternal–infant attachment may not be mediated by mood. The potential relationships between postpartum anxiety-depression, pain, and parenting outcomes deserve further investigation in larger samples because reducing both postpartum pain and improving mood can improve long-term postpartum outcomes.

## Data Availability

Data supporting the findings of this study are available from the corresponding author, but restrictions apply to the availability of these data which were used under license for the current study so are not publicly available. The datasets used and analyzed during the current study available from the corresponding author on reasonable request.
